# Knowledge, attitudes and practices among Australian community pharmacists towards naloxone and overdose prevention: a national cross-sectional study

**DOI:** 10.1007/s11096-026-02219-8

**Published:** 2026-09-05

**Authors:** Louisa Picco, Monica Jung, Rose Laing, Jeffrey Bratberg, Beth Sproule, Suzanne Nielsen

**Affiliations:** 1 Monash Addiction Research Centre, Eastern Health Clinical School, Monash University, Melbourne, Victoria Australia; 2 Department of Pharmacy Practice and Clinical Research, University of Rhode Island College of Pharmacy, Kingston, Rhode Island USA; 3 Centre for Addiction and Mental Health, Toronto, Canada; 4 Leslie Dan Faculty of Pharmacy and Department of Psychiatry, University of Toronto, Toronto, Canada

**Keywords:** Attitudes, Community Pharmacy Services, Harm reduction, Naloxone, Opiate overdose/prevention and control, Pharmacists, Practice

## Abstract

**Introduction:**

Naloxone is an opioid overdose reversal drug that is available for free without a prescription via participating community pharmacies in Australia.

**Aim:**

To investigate Australian community pharmacists’ knowledge, attitudes, practices, and provision of naloxone and overdose prevention, and identify barriers to and correlates of naloxone provision.

**Method:**

Data were collected between January and April 2025 via an anonymous, cross-sectional questionnaire among Australian community pharmacists who were recruited using a randomised representative sampling approach. Data pertaining to pharmacy and pharmacist-related characteristics, pharmacists’ knowledge and attitudes towards naloxone, overdose prevention, and provision of naloxone, including barriers to provision, were captured. Multivariable logistic regression was conducted to determine the pharmacy (including jurisdiction, geographic location, pharmacy type, and provision of other harm reduction services) and pharmacist (including gender, years of experience, knowledge of, and attitudes towards naloxone and overdose prevention) characteristics associated with stocking naloxone.

**Results:**

Of the 730 pharmacists who completed the online questionnaire, approximately three-quarters (n = 534, 73.2%) stocked naloxone. Most pharmacists had positive attitudes towards naloxone (85%), and good or excellent self-reported knowledge (81%) of naloxone and overdose prevention. Logistic regression analyses revealed that pharmacies that had significantly greater odds of stocking naloxone were located in Australia’s least populous states/territories (Australian Capital Territory, Northern Territory, Tasmania, South Australia) (aOR: 2.32; 95% CIs 1.17–4.61) and Queensland (aOR: 2.12; 95% CIs 1.17–3.83), provided Opioid Agonist Treatment (aOR: 2.77; 95% CIs 1.82–4.24), and had pharmacists with good or excellent knowledge of naloxone (aOR: 4.43, CIs 2.75–7.12; aOR: 6.19, CIs 3.53–10.84 respectively) and very positive attitudes towards naloxone supply (aOR: 2.29, 95% CIs 1.23–5.25). Lack of time to develop, implement and/or sustain the program (59.4%) and lack of training among pharmacy staff (44.6%) were identified as key barriers among the minority of pharmacists that did not stock naloxone.

**Conclusion:**

A large proportion of community pharmacies in Australia stocked naloxone, with variability across states/territories. While pharmacists’ attitudes and knowledge were positive overall, there remains scope to increase provision of adequate education and targeted support to strengthen community pharmacies’ harm reduction capacity and provide equitable access.

**Supplementary Information:**

The online version contains supplementary material available at https://doi.org/10.1007/s11096-026-02219-8.

## Impact statements

- Approximately 73% of community pharmacies reported stocking naloxone, with greater odds of stocking naloxone among less populous jurisdictions and pharmacies providing opioid agonist treatment.
- Community pharmacists with greater knowledge of naloxone and more positive attitudes towards naloxone and overdose prevention had significantly higher odds of working in pharmacies that stocked naloxone, highlighting the importance of ongoing education and training.
- Targeted training and education to address jurisdictional differences in service provision and characteristics associated with reduced odds of stocking naloxone could help support greater provision.

## Introduction

Drug-related harms are a global public health concern, with an estimated 27.7 million disability-adjusted life years lost due to drug use in 2021 [1]. Opioids are one of the top contributors to drug-related morbidity and mortality, with almost 100,000 deaths attributed to opioids globally in 2021 [2]. Illicit opioid and non-medical prescription opioid use have been linked with overdose, injection-related injuries or diseases and increased health service utilisation [3, 4]. Naloxone is the recommended first-line treatment for opioid-induced respiratory depression and is a World Health Organisation-listed essential medicine [5, 6]. It can be safely and easily administered via intramuscular injection or nasal spray, with minimal side effects, making it an accessible treatment option for overdose emergencies. This evidence-based harm reduction approach has been demonstrated to effectively reduce opioid-related harms [7, 8]. Globally, major opioid prescribing guidelines, including the 2022 CDC Clinical Practice Guideline for Prescribing Opioids, recommend co-prescription of naloxone, particularly for people at high-risk of opioid overdose [9, 10].

To support reach and uptake of naloxone, many countries, including the US, UK, Canada and Australia, have adopted Take Home Naloxone (THN) Programs, enabling greater access to naloxone for those who may experience or witness an opioid overdose [11–13]. Under these initiatives, naloxone has become more widely accessible through community pharmacies. In Australia, the national THN program was supported by a 4-year $19.6 million federal investment, and from July 2022, naloxone became freely available without a prescription at participating community pharmacies. Pharmacies that opt into the program are required to register online. Once accepted into the program, pharmacists can order naloxone, supply it for free, and claim reimbursement for dispensing it [14]. Over the past decade, these policy changes have been associated with increased naloxone availability in community pharmacies, rising from 23% of pharmacies nationally in 2016 to 60% in 2023 across Australia’s four most populous states [15, 16].

Despite increasing naloxone availability and accessibility in many countries, barriers such as lack of knowledge, poor attitudes and stigma towards the use of opioids and/or naloxone remain [17]. A previous Australian study found that pharmacists were significantly more likely to feel comfortable discussing overdose prevention with patients prescribed opioids compared to those who use illicit opioids [15]. A recent scoping review identified pharmacists’ stigma towards people who inject drugs as a persistent barrier to accessing harm reduction services [18]. Considering the successful implementation of a national THN program in Australia, the identification of correlates of naloxone provision and barriers to access may provide valuable insight to other countries that have yet to implement similar programs. For example, in Germany, THN implementation has been fragmented between states, with no national program, and it is estimated that only 1.3% of those with opioid dependence currently have access to naloxone [19, 20]. Insights from Australia and similar countries such as the US (which, like Germany, are federal systems where different states have their own parliament) could provide key insights into how to nationalise their current approach.

### Aim

To examine Australian community pharmacists’ knowledge, attitudes, practices and provision of naloxone and overdose prevention, and identify barriers to and correlates of naloxone provision.

## Method

### Study design, participants and setting

We conducted an anonymous online survey of Australian community pharmacists, recruiting pharmacists across all states and territories between January and April 2025, using an established randomised representative sampling approach [15, 16], with the aim of recruiting 10% of pharmacies within each state/territory. Detailed recruitment and data collection procedures are published elsewhere [21]. Briefly, two Australian community pharmacy lists obtained from Australian Marketing Lists and Maven Marketing were merged, with duplicates removed. The list was then split by state and territory and randomised. Pharmacies were approached via telephone and the pharmacist-in-charge at the time was asked whether they would be willing to participate in the study and complete an online questionnaire. To avoid issues with cluster bias, one pharmacist-in-charge was invited per pharmacy and provided their email address so the questionnaire link could be directly sent. Follow up emails were sent 1- and 2-weeks after the initial email to encourage participation. This study was reported according to the Strengthening the Reporting of Observational Studies in Epidemiology (STROBE) checklist (Supplementary Material 1).

### Measures

Questionnaire items were designed based on current literature [22, 23], using established questions from previous studies among pharmacists [15, 16] with topics chosen in consultation with an advisory board, which met on three occasions throughout the study and comprised 12 national and international experts in pharmacy harm reduction services.

#### Pharmacist characteristics

Demographic information included pharmacists’ gender and years of professional experience.

#### Pharmacy characteristics

Pharmacy-level characteristics included the pharmacy jurisdiction, location (i.e. capital city/other urban area/rural/remote), type (i.e. chain/banner group or single independent), and provision of other harm reduction services (i.e. opioid agonist treatment (OAT), needle and syringe program).

#### Knowledge about naloxone and opioid overdose

Five items from the validated Opioid Overdose Knowledge Scale (OOKS) [24] were used to measure knowledge about naloxone administration and overdose [16]. Items asked about the length of time it takes for naloxone to have an effect and how long the effects last, where to administer intramuscular naloxone, whether multiple doses of naloxone can be administered and how the effect of naloxone compares to the effect of heroin or methadone.

#### Attitudes towards naloxone and overdose prevention

Pharmacists rated their agreement with statements on a 5-point Likert scale (strongly disagree to strongly agree) which included: (i) overdose prevention for people who use opioids is a waste of time and money; (ii) pharmacists have a role to play in injury prevention, including overdose prevention (iii) training people to identify and respond to an overdose sends the message that misusing pain medications and using heroin is okay, (iv) overdose reversal with naloxone encourages inappropriate use of opioids and (v) offering naloxone in pharmacies is a good use of time and money.

#### Naloxone provision and practice

Pharmacists working in pharmacies that stocked naloxone were asked about formulations stocked, quantity held, frequency of supply in the past three months, any difficulties ordering stock in the past year, and whether their pharmacy is signed up to the THN Program [14].

#### Barriers to stocking naloxone

Pharmacists not stocking naloxone were asked to indicate their reasons, with response options including lack of time to develop, implement, and/or sustain the program, lack of training among pharmacy technicians to implement the program and not enough profit margins.

### Data analysis

We examined pharmacy and pharmacist-level characteristics, forms of naloxone stocked, how often naloxone was supplied in the past three months, and the reasons for not stocking naloxone using descriptive statistics. The five knowledge items from the OOKS were recoded into binary responses (correct response=1 vs incorrect response=0) and were summed to give a total score (0–5), with higher scores indicating greater knowledge. The total scores were then categorised into three groups to reduce sparse data issues in the regression models: scores of 0–2 = “Poor knowledge”, 3–4 = “Good knowledge, and 5 = “Excellent knowledge”. Similarly, the five attitude items were recoded, where the most negative response option was coded − 2, and the most positive response option was coded +2, with some variables reverse-coded. Scores were then summed into a total attitude score of − 10 to +10, with higher scores reflecting more positive attitudes. Scores were consolidated into three categories to facilitate meaningful interpretation of the regression output: − 10 to 0 were coded “negative attitudes”, scores of 1 to 5 were coded “positive attitudes” and scores of 5 to 10 were coded “very positive attitudes”.

Multivariable logistic regression was used to determine the predictors of stocking naloxone, with the adjusted model including pharmacy-level variables (including jurisdiction, geographic location and pharmacy type), pharmacist characteristics (including gender, years of experience, knowledge of, and attitudes towards naloxone) and the provision of other harm reduction services (provision of OAT or needle and syringe program). We tested for multicollinearity between variables in the regression model using the variance inflation factor (VIF<2); no significant interaction was observed. All data analyses were conducted using StataCorp STATA, version 18 BE [25].

### Ethics approval

Ethical approval was granted by the Monash University Human Research Ethics Committee (#45169) on 17 December 2024.

## Results

### Sample characteristics

**Fig. 1:**
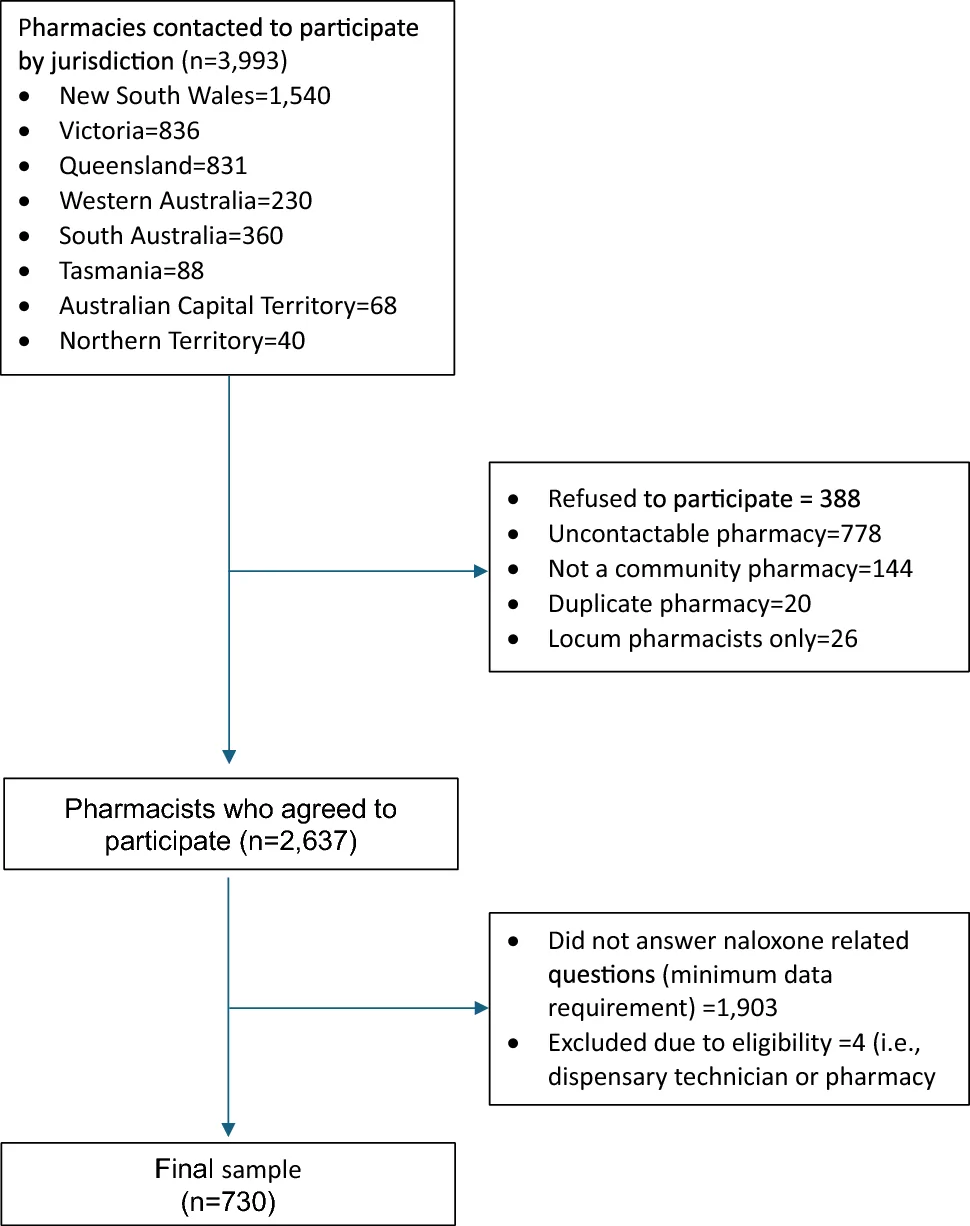
Sample recruitment

In total, 3993 pharmacists were contacted, of whom 2637 agreed to have the questionnaire link sent to their provided email. Of these, 1907 people either did not commence the survey, did not meet the minimum data requirement or were ineligible, with the remaining 730 participants included in the study, giving a response rate of 27.7% (See Fig. 1). The sample represents between 11 and 20% of pharmacies per jurisdiction and was proportional to the number of pharmacies in each jurisdiction (Fig. 1). The sample comprised 53.0% (n = 387) females, and 44.5% (n = 325) had 15 or more years of professional experience in community pharmacy practice. The largest proportion of pharmacies were in capital cities (n = 293, 40.1%), with most pharmacies located in New South Wales (n = 229, 31.4%), Victoria (n = 174, 23.8%) or Queensland (n = 149, 20.4%), Australia’s most populous states (Table 1).

**Table 1:** Pharmacy and pharmacist characteristics associated with stocking naloxone

	Stock naloxone		Total n = 730 (%)
	Yes n = 534 (73.2%)	No n = 196 (26.8%)	
Pharmacy characteristics			
State/territory			
New South Wales	153 (66.8)	76 (33.2)	229 (31.4)
Victoria	122 (70.1)	52 (29.9)	174 (23.8)
Queensland	114 (76.5)	35 (23.5)	149 (20.4)
Western Australia	61 (79.2)	16 (20.8)	77 (10.6)
Australian Capital Territory, Northern Territory, Tasmania, South Australia	84 (83.2)	17 (16.8)	101 (13.8)
Pharmacy location			
Capital city	208 (39.0)	85 (43.4)	293 (40.1)
Urban/Rural/Remote	326 (61.0)	111 (56.6)	437 (59.9)
Pharmacy type			
Chain/banner	338 (74.5)	116 (25.6)	454 (62.2)
Single/independent	196 (71.0)	80 (29.0)	276 (37.8)
Offer Opioid Agonist Treatment			
Yes	264 (52.5)	53 (29.0)	317 (46.2)
No	239 (47.5)	130 (71.0)	369 (53.8)
Offer Needle and Syringe Program			
Yes	262 (56.0)	69 (39.4)	331 (51.5)
No	206 (44.0)	106 (60.6)	312 (48.5)
Pharmacist characteristics			
Pharmacist gender^#^			
Male	247 (72.7)	93 (27.4)	340 (46)
Female	285 (73.6)	102 (26.4)	387 (53)
Years of experience			
<15 years	303 (74.8)	102 (25.2)	405 (55.5)
≥ 15 years	231 (71.1)	94 (28.9)	325 (44.5)

^#^Less than 0.5% indicated “non-binary/gender diverse” or “prefer not to say” and were excluded from analysis

Missing data due to non-response: offer Opioid Agonist Treatment 6.0%; offer Needle and Syringe Program 11.9%; offer OAT 6.0%; offer Needle and Syringe Program 11.9%

### Knowledge about naloxone and opioid overdose

In total, 687 pharmacists completed questions relating to knowledge of naloxone. The mean score for knowledge of overdose was 3.6 (SD 1.39, min 0, max 5). More than a quarter (n = 201, 29.3%) of pharmacists had ‘excellent’ knowledge of naloxone, answering all knowledge questions correctly. Just over half (n = 312, 51.7%) had ‘good’ knowledge, and around 1 in 5 (n = 131, 19.1%) had ‘poor’ knowledge (Table 2).

**Table 2:** Knowledge of naloxone and overdose (n = 687)

	Response options	n (%)
How long does naloxone take to start having an effect?	2–5 min^a^	590 (85.9)
	6–10 min	29 (4.2)
	11–20 min	^
	21–40 min	^
	Don’t know	60 (8.7)
Where is the most recommended place for non-experts to administer IM naloxone?	Outside of thigh or upper arm^a^	580 (84.4)
	Any vein	^
	Heart	0 (0)
	By mouth	11 (1.6)
	Don’t know	91 (13.3)
How long do the effects of naloxone last?	Less than 20 min	99 (14.4)
	About an hour^a^	347 (50.5)
	1 to 6 h	49 (7.1)
	6–12 h	^
	Don’t know	183 (26.6)
If the first dose of naloxone has no effect a second dose can be given	True^a^	527 (76.7)
	False	21 (3.1)
	Don’t know	139 (20.2)
The effect of naloxone is shorter than the effect of heroin or methadone	True^a^	402 (58.5)
	False	36 (5.2)
	Don’t know	249 (36.2)

Responses received for n = 687 pharmacists (missing data = 5.9% of total sample)

^a^Correct response option

^:values not reported where fewer than ten pharmacists responded to an item to maintain anonymity

### Attitudes towards naloxone and overdose prevention

Overall, 698 pharmacists completed questions related to attitudes towards naloxone and overdose prevention. Most pharmacists had positive (n = 312, 44.7%) or very positive (n = 310, 44.4%) attitudes. Very few pharmacists (n = 39, 5.6%) agreed with the statement ‘overdose prevention for people who use opioids is a waste of time and money’, while four in five (n = 576, 82.5%) agreed that ‘pharmacists have a role to play in injury prevention, including overdose prevention’ (Table 3). When compared to responses from a previous 2016 survey, attitudes were shown to be significantly more positive (*p* < 0.001 for all items) (see Supplementary Table 2).

**Table 3:** Pharmacists’ attitudes towards naloxone and overdose prevention (n = 698)

	Strongly disagree n(%)	Disagree n(%)	Neither agree nor disagree n(%)	Agree n(%)	Strongly Agree n(%)	Mean score (range − 2 to +2) (SD)
Overdose prevention for people who use opioids is a waste of time and money^a^	357 (51.15)	231 (33.09)	71 (10.17)	31 (4.44)	8 (1.15)	1.29 (0.90)
Training people to identify and respond to an overdose sends the message that misusing pain medications and using heroin is okay^a^	278 (39.83)	218 (31.23)	73 (10.46)	79 (11.32)	50 (7.16)	0.85 (1.26)
Pharmacists have a role to play in injury prevention, including overdose prevention	23 (3.30)	29 (4.15)	70 (10.03)	331 (47.42)	245 (35.10)	1.07 (0.95)
Overdose reversal with naloxone encourages inappropriate use of opioids^a^	226 (32.38)	254 (36.39)	151 (21.63)	47 (6.73)	20 (2.87)	0.89 (1.03)
Offering naloxone in pharmacies is a good use of time and money	27 (3.87)	18 (2.58)	153 (21.92)	271 (38.83)	229 (32.81)	0.94 (1.00)

Missing data across all variables n = 32. Response options were coded as strongly disagree = − 2, disagree = − 1, neither agree nor disagree = 0, agree = 1 and strongly agree = 2.

^a^ Variables were reverse-coded so that higher scores were associated with positive attitudes toward naloxone and overdose prevention

### Naloxone provision and correlates of stocking naloxone

Three-quarters of pharmacies stocked naloxone (n = 534, 73.2%), of which the majority (n = 443, 83.0%) indicated that their pharmacy is signed up to the national THN Program. Further details on stocking practices are presented in Supplementary Table 3. Multivariable logistic regression revealed that pharmacies located in Australia’s least populous states (Australian Capital Territory, Northern Territory, Tasmania and South Australia) and Queensland were more likely to stock naloxone when compared to pharmacies in New South Wales (adjusted odds ratio (aOR): 2.32; 95% Confidence Intervals (CIs) 1.17–4.61; aOR: 2.12; 95% CIs 1.17–3.83). Pharmacies that offered OAT had significantly greater odds of stocking naloxone (aOR: 2.80; 95% CIs 1.83–4.26) compared to those not providing OAT. Pharmacists with ‘good’ and ‘excellent’ knowledge were four and six times more likely to stock naloxone than those with poor knowledge, respectively (aOR: 4.43, CIs 2.75–7.12; aOR: 6.19, CIs 3.53–10.84). Those with ‘very positive’ attitudes were over twice as likely to stock naloxone when compared with those who had negative attitudes (aOR: 2.29, CIs: 1.23–4.26) (Table 4).

**Table 4:** Correlates of stocking naloxone (n = 640)

Characteristics	Unadjusted odds ratio	Adjusted odds ratio^&^ 95% Confidence Intervals
Pharmacy characteristics		
State/territory		
New South Wales		Reference
Victoria	1.165 (0.761, 1.783)	1.406 (0.845, 2.339)
Queensland	**1.618 (1.013, 2.584) **^**a**^	**2.115 (1.167, 3.834)** ^a^
Western Australia	**1.894 (1.023, 3.504) **^**a**^	1.818 (0.847, 3.902)
Australian Capital Territory, Northern Territory, Tasmania, South Australia	**2.454 (1.361, 4.425) **^**a**^	**2.32 (1.169, 4.605)** ^a^
Pharmacy location		
Capital city		Reference
Urban/ rural/remote	1.200 (0.861, 1.672)	0.871 (0.578, 1.313)
Pharmacy type		
Chain/banner		Reference
Single independent	0.841 (0.602, 1.175)	0.869 (0.578, 1.307)
Offer Opioid Agonist Treatment		
Yes	**2.709 (1.882, 3.900) **^**b**^	**2.795 (1.833, 4.262)**^**b**^
No	Reference	Reference
Offer Needle and Syringe Program		
Yes	**1.954 (1.372, 2.783) **^**b**^	1.343 (0.865, 2.083)
No	Reference	Reference
Pharmacist demographics, knowledge and attitudes		
Gender		
Male		Reference
Female	1.052 (0.757, 1.461)	1.111 (0.754, 1.639)
Years of experience as a pharmacist		
<15 years		Reference
>15 years	0.827 (0.596, 1.149)	0.898 (0.605, 1.334)
Knowledge of naloxone		
Poor knowledge (score of 0–2)		Reference
Good knowledge (score of 3–4)	**4.075 (2.663, 6.236) **^**b**^	**4.428 (2.752, 7.122) **^**b**^
Excellent knowledge (score of 5)	**5.260 (3.197, 8.653) **^**b**^	**6.185 (3.530, 10.839) **^**b**^
Attitudes to naloxone provision		
Negative attitudes (score of − 10-0)		Reference
Positive attitudes (score of 1–5)	1.446 (0.861, 2.428)	1.513 (0.831, 2.755)
Very positive attitudes (score of 6–10)	**2.775 (1.616, 4.763) **^**b**^	**2.290 (1.233, 4.255) **^**a**^

^&^Adjusted for all variables listed in the table

Bold values indicate statistical significance at *p* < 0.05

^a^Significant at the p≤0.05 level. ^b^Significant at the *p* < 0.001 level

Missing data were handled using listwise deletion; due to missing data, 12.3% of the sample were excluded in this analysis

### Reasons for not stocking naloxone

Among the 196 (26.8%) pharmacies that did not stock naloxone, the most commonly reported reasons were (i) lack of time to develop, implement and/or sustain the program (59.4%), (ii) lack of training among pharmacy technician staff to implement the program (44.6%), (iii) lack of knowledge regarding my state’s law and regulations regarding naloxone (36.0%), and (iv) lack of reimbursement mechanism for patient education and counselling (33.1%) (Table 5).

**Table 5:** Reasons for not stocking naloxone (n=175)^$^

Reasons^#^	n (%)
Lack of time to develop, implement, and/or sustain the program	104 (59.4)
Lack of training among pharmacy technician staff to implement the program	78 (44.6)
Lack of knowledge regarding my state’s law and regulations regarding naloxone	63 (36.0)
Lack of reimbursement mechanism for patient education and counselling	58 (33.1)
Not enough profit margins ^^^	43 (24.6)
Lack of support from manager or pharmacy owner	37 (21.1)
Complications with payment	33 (18.9)
Concerns over clientele who might frequent the pharmacy if a program were in place	33 (18.9)
Packing or stocking the various forms of naloxone	31 (17.7)
Moral or ethical concerns associated with drug use and overdose	13 (7.4)
Community opposition	9 (5.1)

^#^Multiple responses could be selected

^$^175/196 pharmacists who work in a pharmacy that don’t supply naloxone responded to this item

^ The National Take Home Naloxone Program reimburses pharmacies for the cost of naloxone, in addition to a small fixed dispensing fee to the pharmacy

## Discussion

This is the first Australian study to comprehensively explore knowledge, attitudes and practices relating to naloxone and overdose prevention, using a nationally representative sampling approach since the implementation of the national THN Program. Overall, pharmacists’ knowledge about and attitudes towards naloxone and overdose prevention were positive, and approximately three-quarters of pharmacies now stock naloxone, with provision increasing by 50 percentage points (representing a nearly 200% relative increase) over the past 10 years.

Pharmacists generally had good naloxone knowledge, which positively correlated with stocking naloxone. When compared to a 2016 national study among community pharmacists, mean naloxone knowledge scores increased twofold from 1.8 to 3.6 (total score out of 5) [16] (Supplementary Material Fig. 1). This is likely explained by several factors, including the implementation of the national THN Program in 2022 [14], alongside extensive promotion and education initiatives, particularly for pharmacists. Other factors, including the implementation of real-time prescription drug monitoring programs (between 2019 and 2024) and the introduction of smaller opioid pack sizes (2020), may have also contributed to enhanced pharmacists’ knowledge. More recently, the Opioid Safety Toolkit (a digital platform providing information on opioid safety, overdose prevention, and practical resources) was launched in 2024 and was promoted nationally throughout community pharmacies [26, 27]. Pharmacists’ knowledge may reflect Australia’s policy and educational context and may not be fully generalisable internationally. However, the association between knowledge and naloxone provision is likely to be applicable to other settings, as studies from the US and Canada have similarly identified education and training as key facilitators of naloxone supply [28, 29].

Pharmacists with positive attitudes towards naloxone and overdose prevention were more likely to stock naloxone, with attitudes becoming significantly more positive over the past decade [16]. This may be attributed to greater awareness of opioid-related risks and harms. However, pharmacists’ attitudes appeared more neutral regarding some statements, including ‘overdose reversal with naloxone encourages inappropriate use of opioids’ and ‘offering naloxone in pharmacies is a good use of time and money’. This reflects similar patterns in attitudes observed within the Australian general population. Between 2016 and 2023, support for naloxone has risen and opposition has declined [30]. While neutral or negative pharmacist attitudes were less common, these perspectives may still prevent naloxone provision and the initiation of conversations around opioid safety and overdose prevention. Pharmacists’ stigma towards people who inject drugs is well-documented globally [31, 32]. Education is a proven method to reduce stigma towards people who inject drugs, and previous studies from the US have found a positive correlation between the provision of education and improved pharmacists’ attitudes towards the provision of harm reduction services [33, 34]. Evidence-based strategies for addressing stigma around people who use drugs could include enhanced education and structured contact with people with lived experience to promote positive attitudes and proactive engagement in naloxone provision and education during routine pharmacy practice [34, 35].

Naloxone provision has increased steadily over the past decade, with approximately three-quarters of Australian community pharmacies (73.2%) currently stocking naloxone, compared to just 23% in 2016 [15, 16, 36]. However, odds of stocking naloxone varied significantly between jurisdictions, with less populous states (i.e. Queensland, Australian Capital Territory, Northern Territory, Tasmania, and South Australia) having greater odds of stocking than in larger and more populous states. While the federally funded THN program operates at a national level, the policy is implemented and regulated at a state level, which may contribute to these jurisdictional differences [37]. Pharmacies that offered OAT also had higher odds of stocking naloxone, which is consistent with previous research [15, 36]. These findings suggest that pharmacists who routinely engage with patients at risk of opioid-related harm may be more attuned to overdose prevention, including providing naloxone [16]. Pharmacies offering a needle and syringe program did not see the same impact with odds of stocking naloxone not being significantly higher among this group.

Pharmacists not stocking naloxone reported lack of time, training and knowledge to be the most common barriers to stocking naloxone. Studies in the US showed that pharmacist interventions and overdose risk training increased naloxone dispensing and willingness to initiate dispensing [38, 39], with evidence suggesting that knowledge about prescription overdose and skills to provide overdose counselling should be core competencies within pharmacy courses [29]. Approximately a third of pharmacists (33.1%) indicated that a lack of reimbursement prevented service uptake. These findings are consistent with previous qualitative studies which identified financial costs associated with training and staffing, and the set price of naloxone [8, 40]. This may indicate that reimbursement through the THN program may be perceived as insufficient to adequately support service provision.

### Strengths and limitations

Strengths of this study include utilising a nationally representative sampling approach to recruit a sample of Australian community pharmacists, with characteristics broadly consistent with those reported in the Australian Health Practitioner Regulation Agency (AHPRA) report [41]. Additional strengths include the nationwide scope, capturing data across all jurisdictions and rurality and the utilisation of well-established recruitment methods and questionnaire measures [16, 42]. The following limitations should be considered. The questionnaire relied on self-reported data, which may be subject to recall or response bias, although such data are commonly used in health service research and can indicate broader trends in naloxone provision [43]. Social desirability bias on attitude-related items may have occurred but was minimised through anonymity. Some items combined multiple barriers within the one response option (e.g. lack of time to develop, implement, and/or sustain the program), limiting the ability to assess their individual contributions. The current findings may not be generalisable to other countries, however, similarities with systems in other countries, such as the UK and Canada, suggest identified patterns and barriers may still be relevant internationally [44]. It is possible that busier or higher-volume pharmacies may have declined or were uncontactable. However, as higher-volume Australian pharmacies are typically part of chain/banner groups, and our sample aligns with national estimates, this was unlikely to significantly affect the findings [45].

## Conclusion

Over the past decade, pharmacists’ naloxone knowledge has increased significantly, with most community pharmacies now stocking naloxone, with variability across jurisdictions. Negative pharmacists’ attitudes towards naloxone may indicate a lack of knowledge and stigma towards people who use drugs, highlighting an important area for intervention. Targeted support to implement the provision of naloxone service within community pharmacies and adequate staff training may be critical for continued service provision and strengthening the broader harm reduction capacity within community pharmacies.

## Supplementary Information

Below is the link to the electronic supplementary material.

Supplementary file1 (DOCX 53 KB)

## Data Availability

The datasets generated and analysed during the current study are available from the corresponding author upon reasonable request.
